# The Landscape of Genomic Alterations in Receptor Tyrosine Kinase Pathways in Biliary Cancers: Implications for Targeted Therapies

**DOI:** 10.1007/s12029-025-01335-4

**Published:** 2025-10-18

**Authors:** Ioannis A. Voutsadakis

**Affiliations:** 1 Holden Comprehensive Cancer Center, University of Iowa Hospitals and Clinics, 200 Hawkins Dr. , Iowa City, IA 52240 U.S.A.; 2 University of Iowa Carver College of Medicine, Iowa City, IA U.S.A.; 3 Division of Clinical Sciences, Section of Internal Medicine, Northern Ontario School of Medicine, Sudbury, ON Canada

**Keywords:** Cholangiocarcinoma, Gallbladder carcinoma, Receptor tyrosine kinases, FGFR, Targeted therapy, Genomic alterations

## Abstract

**Background:**

Biliary carcinomas are aggressive cancers with a high mortality rate. When metastatic, biliary cancers are associated with a short survival and low response to treatments. The first line therapy of metastatic biliary carcinomas consists of a platinum doublet chemotherapy combination with an immune checkpoint inhibitor and results in a median overall survival in the range of approximately 12–13 months, with 20% to 25% of patients surviving at 2 years. Second line chemotherapy options based on fluoropyrimidines are associated with a median survival of less than 6 months. Genomic studies in recent years have clarified molecular aspects of biliary cancers and have confirmed the molecular heterogeneity between the intrahepatic, extrahepatic and gallbladder primary sites.

**Methods:**

Publicly available genomic cohorts of biliary cancer primary locations were interrogated for common mutations and copy number alterations with a focus on receptor tyrosine kinases and their signal transduction pathways.

**Results:**

Specific mutations and structural alterations have different prevalence depending on the primary location. Alterations in receptor tyrosine kinases and the transduction pathways originating from them show differential prevalence in the primary locations of the biliary cancers and create diverse treatment opportunities that can be harnessed for drug development. Approximately 49% of intrahepatic, 57.6% of gallbladder, and 66% of extrahepatic carcinomas harbor RTK pathway alterations.

**Conclusions:**

Targeted therapies for individual components of these kinase receptors and pathways, including FGFR2, HER2, BRAF and others, have already been introduced in clinical practice for the treatment of patients with biliary tumors bearing alterations in these genes. The findings underscore the need for primary site-driven genomic testing to guide therapy selection. The current analysis discusses strategies to create opportunities for clinically available targeted therapies.

## Introduction

Biliary cancers are less prevalent than other gastrointestinal tract carcinomas, such as colorectal and gastric, but they are highly lethal [1]. Liver cancers (predominantly hepatocellular carcinomas) account for about 865,000 annual cases, globally, with intrahepatic cholangiocarcinoma (CCA) representing less than 15% of these, while gallbladder cancers add about 120,000 cases annually [1]. Biliary cancers are categorized according to their primary location as intrahepatic, extrahepatic and gallbladder carcinomas. Some classifications consider carcinomas of the ampulla of Vater/peri-ampullary carcinomas to be a fourth category of biliary carcinomas, although tumors of this location are histologically either of a pancreatobiliary sub-type or of a duodenal intestinal sub-type [2]. Intrahepatic cholangiocarcinomas may be derived from small ducts or from large ducts and extrahepatic cholangiocarcinomas can be divided according to their location to perihilar and distal [3]. Extrahepatic cholangiocarcinoma (eCCA) is anatomically and therapeutically subdivided into perihilar CCA (pCCA/Klatskin tumor), arising at the hepatic duct bifurcation and representing 50–60% of all CCAs, and distal CCA (dCCA), occurring in the common bile duct and representing 20–30% of all CCAs. Consequently, among CCAs, eCCA (pCCA and dCCA) represents 70% to 90% of cases, while intrahepatic CCA (iCCA) comprises only 10% to 20%. Gallbladder carcinomas constitute about 20% to 25% of all biliary tract carcinomas. This distinction critically impacts clinical management as pCCA often necessitates complex biliary drainage procedures when unresectable, while dCCA may require pancreaticoduodenectomy, when resectable. While FGFR2 and IDH1-targeted therapies are approved specifically for iCCA, tumor-agnostic agents (e.g., anti-HER2 targeted agents for *ERBB2* amplifications, BRAF inhibitors for *BRAF* V600E mutations) benefit eligible eCCA/gallbladder carcinoma patients. Risk factors for the development of biliary cancers include cholelithiasis, specifically for gallbladder carcinoma, primary sclerosing cholangitis, steatohepatitis and obesity [4]. Viral hepatitis is a risk factor especially for iCCAs. Parasitic infections are less common causes in western populations but they play a significant role in certain other geographic locations in the East, especially for eCCAs. In many cases no identifiable risk factors for the development of biliary carcinomas are present [4]. Epidemiologic variability in biliary cancers of the different primary locations exists in gender and geographic distribution. Gallbladder carcinomas, for example, are more frequent in women, with a 2 to 1 prevalence compared to men, suggesting a role for sex hormones and reproductive history [5]. The gallbladder biliary cancer primary location has also the highest prevalence of HER2 alterations. Gallbladder carcinomas are most prevalent in some geographic hotspot areas such as the northeastern India and south Chile [6]. More than half of the global cases of gallbladder carcinomas are diagnosed in eastern and south Asia [6]. In contrast, iCCAs are male predominant and show more frequent IDH and FGFR2 alterations.

Progress in treatment and better outcomes in biliary cancers rely on an accurate differential diagnosis from other frequent cancers of the region, such as hepatocellular and pancreatic carcinomas. Intrahepatic cholangioacarcinomas can be differentiated from hepatocellular carcinomas on the basis of morphology and immunohistochemistry, with the former being positive for cytokeratins 7 and 19 and the latter being positive for Hepar1 [7]. Alpha fetoprotein is also frequently elevated in hepatocellular carcinomas and may help in the differential diagnosis. The systemic treatment of the two liver carcinoma types are divergent with chemotherapy plus immunotherapy being the first line option in cholangiocarcinomas, while anti-angiogenic targeting with immunotherapy or combination immunotherapies are the preferred systemic treatments for hepatocellular carcinomas [8, 9].

Many targeted treatments arise as useful options in biliary cancers with specific genomic alterations. Although these alterations are individually rare, collectively receptor tyrosine kinase pathway defects occur in 49–66% of biliary tract carcinomas, enabling targeted therapy in most patients. Frequent alterations in biliary cancers occur in the super-family of receptor tyrosine kinases and downstream pathways. In this study, the genomic environment of biliary carcinomas is evaluated with a focus on alterations in receptor tyrosine kinase pathway constituents and a view for current and future therapeutic implications. In addition the study quantifies receptor tyrosine kinase pathway alterations across biliary subtypes (iCCA, eCCA, GBC) and their implications for site-specific targeted therapies.

## Methods

The three primary locations of biliary cancer, intrahepatic, extrahepatic and gallbladder carcinomas were analyzed separately using three publicly available genomic series [10–12]. The intrahepatic cholangiocarcinoma series of MSK (Memorial Sloan Kettering Cancer Center, Intrahepatic cholangiocarcinoma_MSK) included 412 patients [10]. The gallbladder carcinoma cohort analyzed was also from MSK (Gallbladder Cancer_ MSK) and included 233 patients [11]. MSK series use for their mutation analyses an extensive targeted panel of cancer associated genes, called MSK-IMPACT (Memorial Sloan Kettering Integrated Mutation Profiling of Actionable Cancer Targets) that includes 341 to 505 sequenced genes in different versions. MSK-IMPACT versions were harmonized to the smallest common gene set.

Extrahepatic cholangiocarcinomas were procured for the current analysis from the pan-cancer collection of the American Association for Cancer Research (AACR) project GENIE (Genomics Evidence Neoplasia Information Exchange, GENIE Cohort v18.0 public), which includes a cohort of 256 patients [12]. The extensive clinicopathologic and genomic database of project GENIE is a collaboration of several institutions, across the United States and from Canada and France, coordinated by AACR. Different pipelines used in the participating centers were harmonized for combined analysis and presentation by the project. GENIE fusion data were unavailable, limiting alteration comparisons. Ampullary carcinomas were excluded from the analysis due to their distinct molecular profiles from biliary carcinomas and their heterogeneity and segregation in intestinal and pancreatobiliary sub-types [2].

All analyses were performed in cBioCancer Genomics Portal (cBioportal, assessed from www.cbioportal.org), which is an openly available cancer genomics site that harbors openly available genomic data across cancer sub-types [13, 14]. cBioportal hosts curated data that can be easily summarized from the platform, and groups of interest within individual studies can be constructed for separate analyses. All cohorts were institutionally distinct (MSK versus GENIE consortium). No patient overlap existed between intrahepatic and gallbladder MSK cohorts per the source publications. The clinical implications of mutations of interest were assessed based on the OncoKB, a knowledgebase of genomic alterations that lists specific mutations observed in cancer and assigns a functional attribute [15, 16]. OncoKB categorizes cancer-associated mutations as oncogenic or potentially oncogenic, potentially neutral or as variants of unknown significance. High tumor mutation burden (TMB) was defined as more than 10 non-synonymous mutation per megabase (Mb), a cut-off that has been used in clinical trials [17].

### Statistical Methods

Descriptive statistics were calculated for means and medians of the groups. The statistical comparisons of continuous parameters of groups of interest were performed with the Student’s t test, and with the Fisher’s exact test or the χ^2^ test for categorical comparisons. The Fisher's exact test was used for comparisons with expected counts below 5. Elsewhere the χ2 test was applied. Normality was checked with the Shapiro–Wilk test and comparisons of values not normally distributed were made with the Mann Whitney U test. Significance was set at p less than 0.05. The Benjamini–Hochberg procedure was applied for correction of multiple comparisons. All statistical calculations were performed at the QuickCalcs GraphPad online platform (graphpad.com/quickcalcs/) Survival analysis was performed with construction of Kaplan Meier curves which were compared with the Log Rank test using the online Statistics Kingdom site (statskingdom.com/Kaplan–meier). No new code was written for the computational analyses.

### Ethical Statement

As the study included only a secondary analysis of de-identified publicly available data, it was exempt from Institutional Research Board approval, per local regulations.

## Results

### Genomic Landscapes of Intrahepatic Cholangiocarcinoma

Overall 200 patients of total 412 patients (49%) in the intrahepatic cholangiocarcinoma MSK cohort had alterations in one or more of the 19 receptor tyrosine kinase pathway genes examined (*KRAS*, *NRAS*, *BRAF*, *PIK3CA*, *EGFR*, *ERBB2*, *ERBB3*, *ERBB4*, *FGFR1*, *FGFR2*, *FGFR3*, *FGFR4*, *NTRK1*, *NTRK2*, *NTRK3*, *PTEN*, *AKT1*, *AKT2*, *AKT3*) (Table 1). The most prevalent mutations in the pathways were in *KRAS* (19.5% of all patients in the group with receptor tyrosine kinase pathway gene alterations), *NRAS* (6.5%), *BRAF* (13%), and *PIK3CA* (8.5%). In addition, *FGFR2* was altered in 34% of cases with receptor tyrosine kinase pathway gene alterations (28% fusions, 6% mutations). Among cases with *FGFR2* alterations 82.4% were fusions. In the entire intrahepatic cholangiocarcinoma cohort, *FGFR2* alterations were present in 16.5% of cases. FGFR2 fusions, which represent 28% of receptor tyrosine kinases-altered cases, are actionable with FDA-approved inhibitors futibatinib and pemigatinib. Other genes with lower prevalence of alterations include the three NTRK receptor homologues, as well as *ERBB2*, *ERBB3* and *PTEN* (Table 1). Regarding specific *KRAS* mutations, canonical G12 mutations are the most prevalent (29 of 39 total cases with KRAS mutations, 74.4%). The most frequent G12 mutation in intrahepatic cholangiocarcinomas was the G12D substitution (16 cases), followed by G12V (8 cases) and G12C (3 cases). Other less commonly mutated codons was Q61 (5 cases) and G13 (3 cases). Among the 13 cases with *NRAS* mutations present in the series, the most commonly mutated codon was Q61 (8 cases, 6 of them Q61R), followed by G12 (3 cases, 2 of these G12D) and G13 (2 cases). Eleven of 27 mutations (40.7%) in *BRAF* were classic V600E mutations. Twelve additional mutations in neighboring codons or elsewhere in the gene were considered oncogenic. Sixteen of 17 mutations (94.1%) in *PIK3CA* were also oncogenic and 12 of the 17 were in the hotspot codons E545 (5 cases), E542 (4 cases), H1047 (2 cases) and Q546 (one case). Fusions of *FGFR2* involved a variety of partner genes the most prevalent being *BICC1*.

**Table 1:** List of alterations in receptor tyrosine kinase pathway genes in the MSK intrahepatic cholangiocarcinoma series (*n* = 412)

Gene	Alterations (n) (%)	Mutations (n)		amplifications	fusions	treatment
		all	Putative driver			
KRAS	40 (9.7)	39	39	1		Specific inhibitors, e.g. for KRAS G12C
NRAS	13 (3)	13	13			
BRAF	27 (7)	26	23		1	V600E inhibitors combinations with anti-EGFR monoclonal antibodies
PIK3CA	17 (4)	17	16			? PIK3CA inhibitors combinations
EGFR	8 (1.9)	1	0	7		?Anti-EGFR monoclonal antibodies
ERBB2	12 (2.9)	5	2	7		Anti-HER2 therapies
ERBB3	10 (2.4)	6	3	4		?Anti-HER3 monoclonal antibodies
ERBB4	7 (1.7)	7	0			
FGFR1	5 (1.2)	2	0	2 (+ 1 deletion)		Specific inhibitors: Futibatinib, pemigatinib
FGFR2	68 (16.5)	12	10		56	
FGFR3	7 (1.7)	4	0	2	1	
FGFR4	5 (1.2)	1	0	3	1	
NTRK1	12 (2.9)	3	0	7	2	Specific inhibitors: Entrectinib, larotrectinib
NTRK2	1 (0.2)	1	0			
NTRK3	6 (1.5)	6	0			
PTEN	10 (2.4)	10	7			? PI3K or AKT inhibitors
AKT1	2 (0.5)	0	0	2 deletions		?AKT inhibitors
AKT2	1 (0.2)	0	0	1		
AKT3	6 (1.5)	3	0	3		
all	200 (49)	156	113	37 (+ 3 del)	61	

? in the treatment column in front of mentioned treatments denotes that the treatment is experimental or based on data in other cancers with similar alterations. Treatments may not be appropriate for all types of alterations in the respective genes.

Intrahepatic cholangiocarcinoma patients with alterations in receptor tyrosine kinase pathway genes did not differ from patients without such alterations in their average age at presentations, prevalence of early onset (at age younger than 50 years old) cancers, gender distribution, ECOG performance status or relevant comorbidities (Table 2). A higher percentage of patients in the group without alterations in receptor tyrosine kinase pathway genes (36.7%) were obese compared with the group with alterations (26.3%, obesity prevalence difference: 10.4%, 95% Confidence Interval (CI): 1.5%−19.3%, Fisher’s exact test *p* = 0.02). This implies that putative causative molecular pathways related to obesity and the metabolic syndrome have a differing importance. in the two groups. Tumors without alterations in receptor tyrosine kinase pathway genes may rely more often on the hyperinsulinemia present in obesity and the associated metabolic syndrome to activate the downstream pathways in the absence of alterations. Patients with alterations in receptor tyrosine kinase pathway genes presented more frequently with metastatic disease (Metastasis prevalence: 52% versus 34.9%, metastasis prevalence difference: 17.1%, 95% CI: 7.6%–26.5%, Fisher’s exact test *p* = 0.001, Table 3). In contrast, no differences in the tumor grade and the source duct type (large versus small ducts) were observed between the groups (Table 3). The average TMB was higher in the group with receptor tyrosine kinase pathway gene alterations (mean 4.3 non-synonymous mutations per Mb) compared with the group without receptor tyrosine kinase pathway gene alterations (mean 2.9 non-synonymous mutations per Mb, Mann Whitney U test *p* = 0.03). However, the prevalence of high TMB (above 10 non-synonymous mutations per Mb) or the prevalence of high fragment genome altered (FGA) were not different between the groups (Table 3).

**Table 2:** Clinical characteristics of intrahepatic cholangiocarcinoma patients in the entire MSK cohort and in the groups with or without receptor tyrosine kinase pathway gene alterations

	All (*n* = 412)(%)	Patients with receptor tyrosine kinase pathway gene alterations (*n* = 200) (%)	Patients without receptor tyrosine kinase pathway gene alterations (*n* = 212) (%)	p
Age (mean)	62.8 ± 11.9	61.8 ± 11.9	63.7 ± 11.9	0.1
Early onset (≤ 50 years-old)				
yes	59 (14.3)	30 (15)	29 (13.7)	0.77
no	353 (85.7)	170 (85)	183 (86.3)	
NA				
Sex				
Male	284 (64.5)	90 (45)	100 (47.2)	0.69
Female	156 (35.5)	110 (55)	112 (52.8)	
Obesity				
BMI ≤ 30	279 (68.4)	146 (73.7)	133 (63.3)	0.02
BMI > 30	129 (31.6)	52 (26.3)	77 (36.7)	
NA	4	2	2	
ECOG PS				
0	171 (43.1)	86 (44.6)	85 (41.7)	0.82
1	212 (53.4)	100 (51.8)	112 (54.9)	
2–3	14 (3.5)	7 (3.6)	7 (3.4)	
NA	15	7	8	
Smoking status				
Never	202 (49.4)	97 (48.7)	105 (50)	0.33
Former	166 (40.6)	86 (43.2)	80 (38.1)	
Current	41 (10)	16 (8.1)	25 (11.9)	
NA	3	1	2	
Diabetes				
Yes	84 (20.4)	40 (20.1)	44 (20.8)	0.9
No	327 (79.6)	159 (79.9)	168 (79.2)	
NA	1	1	0	
Viral Hepatitis				
Yes	33 (8)	15 (7.5)	18 (8.5)	0.72
No	379 (92)	185 (92.5)	194 (91.5)	
Cirrhosis				
Yes	29 (7.2)	12 (6.2)	17 (8.1)	0.56
No	374 (92.8)	181 (93.8)	193 (91.9)	
NA	9	7	2	

*BMI*: Body Mass Index. *ECOG PS*: Eastern Co-operative Oncology Group Performance Status, *NA*: Not available.

**Table 3:** Tumor clinical, pathologic and genomic characteristics of the entire MSK intrahepatic cholangiocarcinoma cohort and of the groups with and without alterations in receptor tyrosine kinase pathway genes

	All (*n* = 412)(%)	Patients with receptor tyrosine kinase pathway gene alterations (*n* = 200) (%)	Patients without receptor tyrosine kinase pathway gene alterations (*n* = 212) (%)	p
Disease extend				
Solitary	148 (35.9)	58 (29)	90 (42.5)	0.001
Multifocal	86 (20.9)	38 (19)	48 (22.6)	
Metastatic	178 (43.2)	104 (52)	74 (34.9)	
Grade				
Well differentiated	15 (3.8)	7 (3.8)	8 (3.9)	0.2
Moderately differentiated	231 (58.9)	104 (55.6)	127 (62)	
Poorly differentiated	146 (37.3)	76 (40.6)	70 (34.1)	
NA	20	13	7	
Duct category				
Small duct type	176 (88.4)	75 (84.3)	101 (91.8)	0.11
Large duct type	23 (11.6)	14 (15.7)	9 (8.2)	
Indeterminate/not available	213	111	102	
Ca19-9				
≤ 100	179 (57.6)	80 (53)	99 (61.9)	0.13
> 100	132 (42.4)	71 (47)	61 (38.1)	
NA	101	49	52	
TMB				
High (> 10 mutations/Mb)	20 (4.9)	12 (6)	8 (3.8)	0.36
Low (≤ 10 mutations/Mb)	392 (95.1)	188 (94)	204 (96.2)	
FGA				
< 0.1	150 (36.4)	70 (35)	80 (37.7)	0.6
> 0.1	262 (63.6)	130 (65)	132 (62.3)	

*TMB*: Tumor Mutation Burden, *FGA*: Fragment Genome Altered, *NA*: Not available

**Fig. 1:**
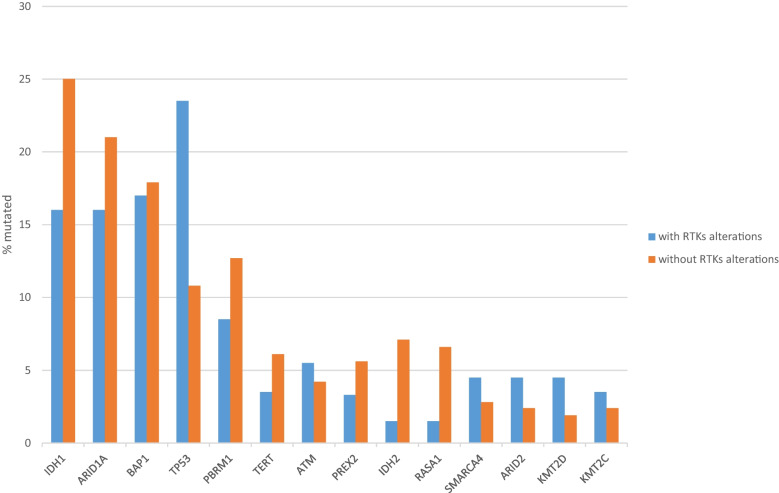
Prevalence of mutations in intrahepatic cholangiocarcinomas with (blue bars) and without (orange bars) receptor tyrosine kinase alterations. Data are from the MSK intrahepatic cholangiocarcinoma series

The most prevalent mutations in intrahepatic cholangiocarcinomas, outside those in receptor tyrosine kinase pathway genes, displayed differences in prevalence in the two groups with or without alterations in receptor tyrosine kinase pathway genes. Mutations in isocitrate dehydrogenase homologues IDH1 and IDH2 were significantly more prevalent in cancers without receptor tyrosine kinase pathway gene alterations (25% and 7.1%, respectively), compared with cancers with receptor tyrosine kinase pathway gene alterations (16% and 1.5%, respectively, Fisher’s exact test *p* = 0.02 and 0.006, respectively, Fig. 1). Of note, IDH mutations occur predominantly in intrahepatic tumors, and are less frequent in other biliary locations, suggesting distinct pathogenesis. The two epigenetic modifier genes of the SWI/SNF complex, *ARID1A* and *PBRM1* were collectively more frequently mutated in intrahepatic cholangiocarcinomas without receptor tyrosine kinase pathway gene alterations than in cancers with such alterations (33.7% versus 24.5%, Fisher’s exact test *p* = 0.03, Fig. 1). However, when considered individually the prevalence of mutations in the two epigenetic modifiers were not significantly different in the two groups (Fisher’s exact test *p* = 0.16 and *p* = 0.2, respectively). In contrast, mutations in tumor suppressor *TP53* were more prevalent in cases with receptor tyrosine kinase pathway gene alterations (23.5% versus 10.8% in cases without receptor tyrosine kinase pathway gene alterations, Fisher’s exact test *p* = 0.0009). *TP53* mutation prevalence differences may not reflect functional p53 loss, as the functional status of p53 has not been formally tested in these cases. However, the OncoKB knowledgebase predicts that all *TP53* mutations in the cohort are likely oncogenic [15, 16]. The difference in the prevalence of *TP53* mutations remained statistically significant after correction for multiple comparisons (corrected *p* = 0.01), while all other comparisons became insignificant.

**Fig. 2:**
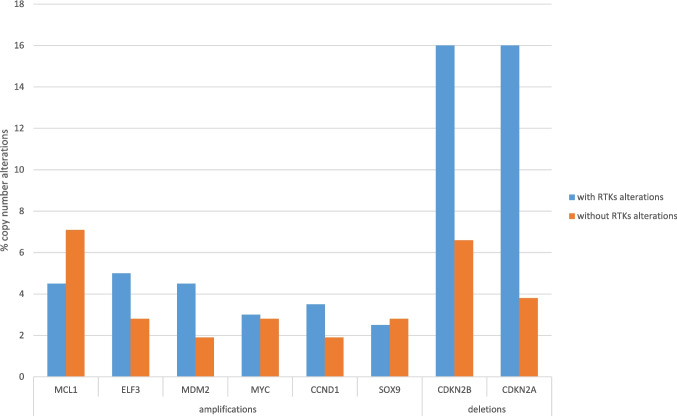
Prevalence of copy number alterations in intrahepatic cholangiocarcinomas with (blue bars) and without (orange bars) receptor tyrosine kinase alterations. Data are from the MSK intrahepatic cholangiocarcinoma series

Among copy number alterations, common amplifications were not significantly different in the two groups. However, deletions of the 9p21.3 locus harboring tumor suppressor genes *CDKN2A* and *CDKN2B* were more prevalent in cases with receptor tyrosine kinase pathway gene alterations (Fisher’s exact test *p* = 0.002 for *CDKN2A* and *p* < 0.0001 for *CDKN2B*, Fig. 2). Both comparisons remained statistically significant after correction for multiple comparisons (corrected *p* = 0.001 and 0.006, respectively). *CDKN2A*/*CDKN2B* losses may sensitize to treatment with CDK4/6 inhibitors, as it will be discussed in a later section on Future Perspectives.

**Fig. 3:**
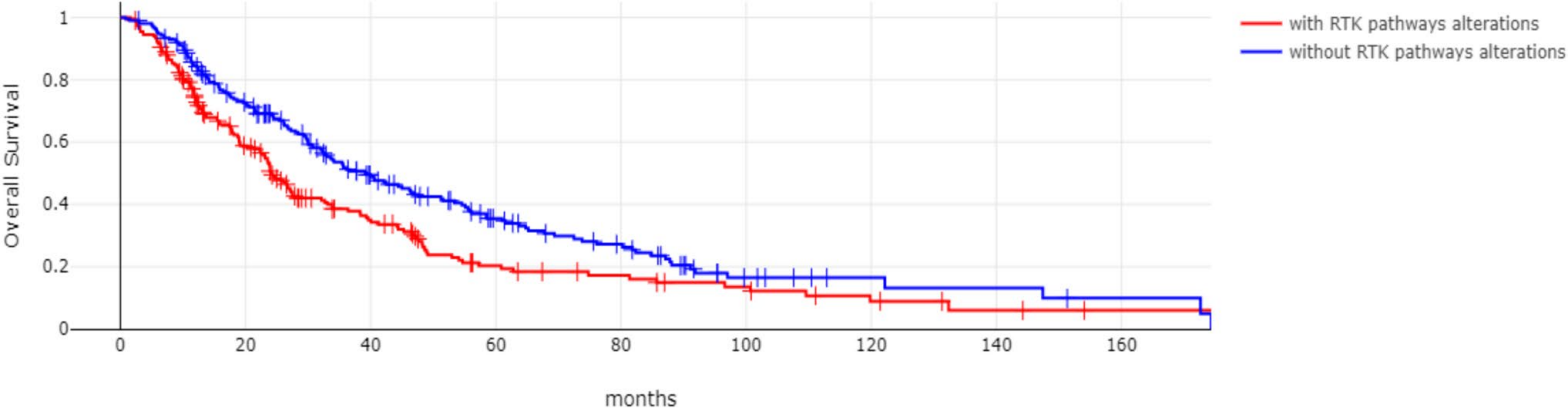
Overall Survival of intrahepatic cholangiocarcinoma patients with (red line) and without (blue line) receptor tyrosine kinase pathway gene alterations. Log Rank *p* = 0.001

**Fig. 4:**
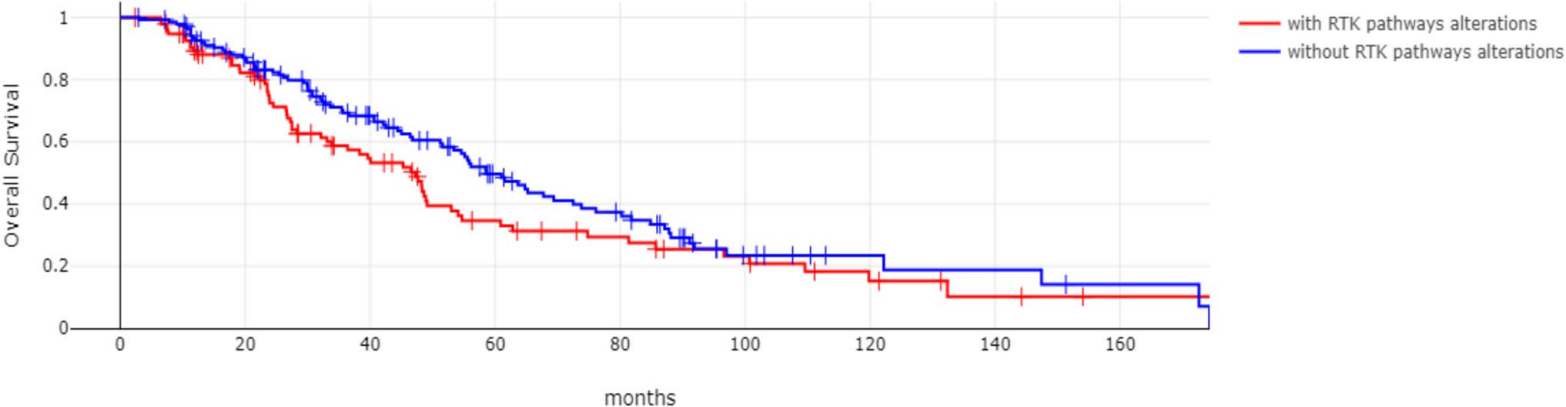
Overall Survival of localized intrahepatic cholangiocarcinoma patients with (red line) and without (blue line) receptor tyrosine kinase pathway gene alterations. Log Rank *p* = 0.08

**Fig. 5:**
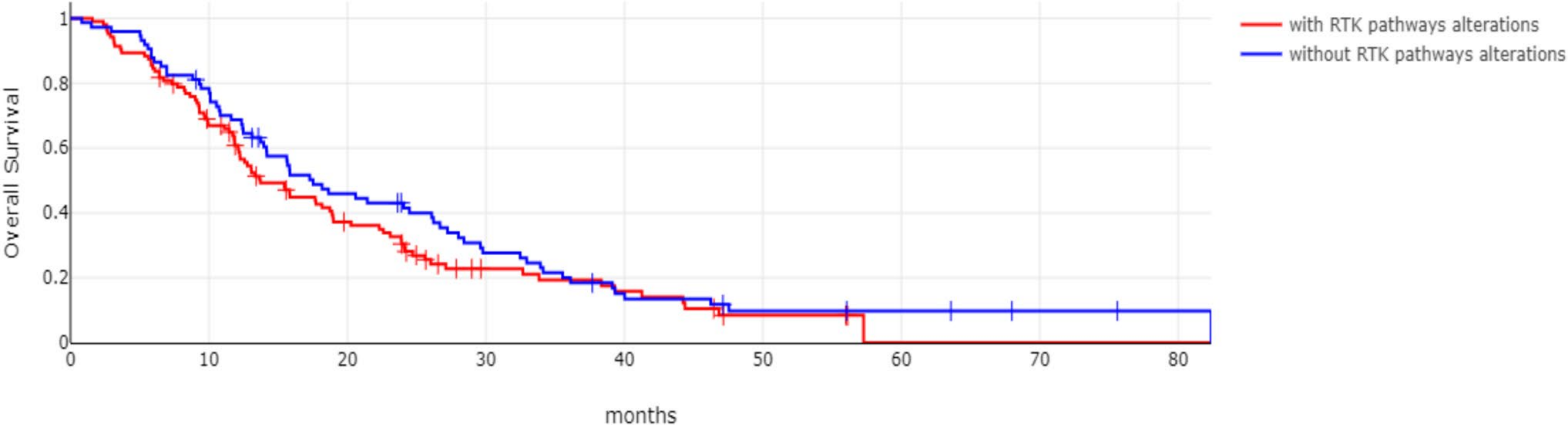
Overall Survival of metastatic intrahepatic cholangiocarcinoma patients with (red line) and without (blue line) receptor tyrosine kinase pathway gene alterations. Log Rank *p* = 0.2

The group of intrahepatic cholangiocarcinoma patients without receptor tyrosine kinase pathway gene alterations had a better Overall Survival (OS) than the group with receptor tyrosine kinase pathway gene alterations (Log Rank test *p* = 0.001, Fig. 3). The group of intrahepatic cholangiocarcinoma patients without receptor tyrosine kinase pathway gene alterations and localized disease had also a better OS, which was, however borderline statistically insignificant (Log Rank test *p* = 0.08, Fig. 4). In addition, the two groups had not significant difference in OS when only patients with metastatic disease were included in the analysis (Log Rank test *p* = 0.2, Fig. 5).

### Genomic Landscapes of Extrahepatic Cholangiocarcinoma

A pure extrahepatic cholangiocarcinoma cohort with 256 samples from 252 patients was available from the project GENIE [12]. In this cohort, 169 of 256 samples (66%) had alterations in one or more of the 19 examined genes of the receptor tyrosine kinase pathways. The most frequent alterations in receptor tyrosine kinase pathway genes were mutations on *KRAS* with a prevalence of 37.9% (Table 4). The highest prevalence of mutations in KRAS were at codon G12 (G12D and G12V and less frequently G12S and the currently targetable G12C mutation), followed by mutations at codon Q61 (most commonly Q61H). All other genes had a lower frequency of mutations. Besides *KRAS*, the only genes of the group with mutations in more than 4% of cases were *ERBB3* (7.1%), *ERBB2* (6.3%), *ERBB4* (5.1%) and *PIK3CA* (4.7%). Amplifications in receptor tyrosine kinase pathway genes were rare, with only *ERBB2* having amplifications in more than 3% of cases (7.2%) among samples with amplification data (*n* = 208). *ERBB2* alterations (13.5% if mutations and amplifications are considered combined) represent a therapeutically actionable subset. Patients in the groups with or without receptor tyrosine kinase pathway genes did not differ in mean age, gender, percentage with high TMB or high FGA (Table 5). GENIE harmonized data across institutions but the platform heterogeneity may limit the TMB and FGA comparability.

**Table 4:** List of alterations in receptor tyrosine kinase pathway genes in the GENIE extrahepatic cholangiocarcinoma series (*n* = 256)

Gene	Alterations (n) (%)	Mutations (n)		Amplifications (*n* = 208) (%)
		all	Putative driver	
KRAS	102 (40.3)	97 (37.9)	96 (37.7)	5 (2.4)
NRAS	6 (2.3)	6 (2.3)	4 (1.6)	0
BRAF	8 (3.2)	7 (2.7)	6 (2.3)	1 (0.5)
PIK3CA	13 (5.1)	12 (4.7)	9 (3.7)	1 (0.5)
EGFR	9 (3.7)	7 (2.7)	2 (0.8)	2 (1)
ERBB2	31 (13.5)	16 (6,3)	10 (4)	15 (7.2)
ERBB3	18 (7.6)	17 (7.1)	7 (2.7)	1 (0.5)
ERBB4	13 (5.1)	13 (5.1)	1 (0.4)	0
FGFR1	3 (1.3)	2 (0.8)	0	1 (0,5)
FGFR2	4 (1.6)	4 (1.6)	1 (0.4)	0
FGFR3	7 (2.9)	4 (1.6)	0	3 (1.4)
FGFR4	3 (1.3)	2 (0.8)	0	1 (0.5)
NTRK1	3 (1.3)	3 (1.3)	0	0
NTRK2	1 (0.4)	1 (0.4)	0	0
NTRK3	4 (1.6)	4 (1.6)	0	0
PTEN	8 (3.2)	7 (2.7)	4 (1.6)	1del (0.5)
AKT1	3 (1.3)	3 (1.3)	1 (0.4)	0
AKT2	1 (0.5)	0		1 (0.5)
AKT3	2 (0.8)	2 (0.8)	0	0
all	169 (66)

**Table 5:** Clinical characteristics of extrahepatic cholangiocarcinoma patients in the GENIE cohort and in the groups with or without receptor tyrosine kinase pathway gene mutations

	All (*n* = 231)(%)	Patients with receptor tyrosine kinase pathway gene mutations (*n* = 155) (%)	Patients without receptor tyrosine kinase pathway gene mutations (*n* = 76) (%)	p
Age (mean)	64.2 ± 12.2	64.4 ± 12.5	63.7 ± 11.8	0.48
Early onset (≤ 50 years-old)				
yes	30 (13)	21 (13.5)	9 (11.8)	0.83
no	201 (87)	134 (86.5)	67 (88.2)	
Sex				
Male	139 (61)	87 (57.2)	52 (68.4)	0.11
Female	89 (39)	65 (42.8)	24 (31.6)	
NA	3	3		
TMB				
High (> 10 mutations/Mb)	28 (12.8)	22 (14.2)	6 (9.5)	
Low (≤ 10 mutations/Mb)	190 (87.2)	133 (85.8)	57 (90.5)	0.5
NA	13	0	13	
FGA				
< 0.1	91 (61.1)	57 (58.2)	34 (66.7)	0.37
> 0.1	58 (38.9)	41 (41.8)	17 (33.3)	
NA	82	57	25	

Data were available for 231 of the 256 patients in the series. *TMB*: Tumor Mutation Burden, *FGA*: Fragment Genome Altered, *NA*: Not available.

**Fig. 6:**
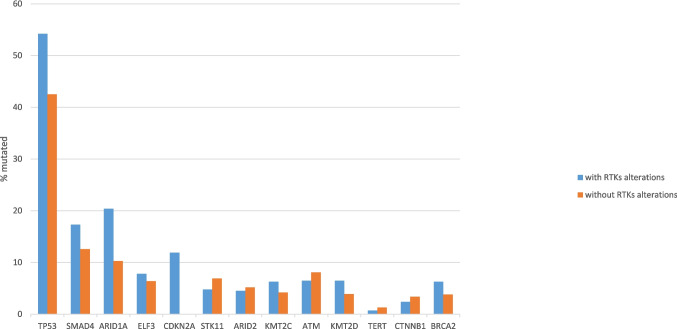
Prevalence of mutations in extrahepatic cholangiocarcinomas with (blue bars) and without (orange bars) receptor tyrosine kinase alterations. Data are from the American Association for Cancer Research (AACR) project GENIE (Genomics Evidence Neoplasia Information Exchange)

Mutations in the tumor suppressor p53 gene *TP53* were prevalent in 50.2% of extrahepatic cholangiocarcinomas with no significant difference in prevalence in tumors with receptor tyrosine kinase pathway alterations (54.2%) and without such alterations (42.5%, Fisher’s exact test *p* = 0.11, Fig. 6). Though non-significant, *TP53* mutations trended higher in receptor tyrosine kinase pathway-altered tumors, mirroring intrahepatic cholangiocarcinomas findings (23.5% vs. 10.8%, *p* < 0.001). The second most prevalent mutations in extrahepatic cholangiocarcinomas were in epigenetic modifier *ARID1A* gene and were more prevalent in cases with receptor tyrosine kinase pathway alterations (20.4%) while their prevalence was 10.3% in cases without such alterations (*ARID1A* mutations: 20.4% versus 10.3%, OR 2.3, 95% CI: 1.0–5.2, Fisher’s exact test *p* = 0.04). This difference remains exploratory as it was not significant after correction for multiple comparisons (Benjamini–Hochberg adjusted *p* = 0.17). Mutations in the gene encoding for the transducer of the TGFβ pathway SMAD4 were observed in 15.7% of extrahepatic cholangiocarcinomas and showed no differences in the groups with or without receptor tyrosine kinase pathway alterations (Fisher’s exact test *p* = 0.37, Fig. 6). SMAD4 loss of function disrupts TGFβ signaling and mutations have been linked with improved progression free survival and overall survival in advanced biliary cancers [18]. Other cancer-associated mutations were less prevalent with an overall frequency of less than 10% and no significant differences in the groups with or without receptor tyrosine kinase pathway alterations (Fig. 6). Copy number alterations were also prevalent in less than 10% of cases with no significant differences observed in the two groups, contrasting with gallbladder carcinoma CDK12 and CDK4 amplifications. Fusions were not assessed in the GENIE cohort.

### Genomic Landscape of Gallbladder Carcinoma

The gallbladder carcinoma cohort of the MSK study included 233 patients (244 samples) and 136 samples (57.6%) had one or more alterations in the 19 examined genes of the receptor tyrosine kinase pathways (Table 6). In the case of gallbladder carcinomas, the most prevalent mutations in genes of the receptor tyrosine kinase pathways were in *PIK3CA* (18.4% (25 of 136) of all patients in the group with receptor tyrosine kinase pathway gene alterations), *KRAS* (12.5%, of note *KRAS* displays additionally amplifications in 7.4% of cases, see later in the paragraph), and in two members of the EGFR family, *ERBB2* (11.8%) and *ERBB3* (10.3%). The most frequent *PIK3CA* mutations were E542K (6 of 30 cases 20%) followed by mutations at codons E545 and H1047 (5 cases each). KRAS mutations were most frequently in G12 codon (10 of 18 cases, 55.5%, of which 5 were G12D and two cases each, 11.1%, were G12A and G12C). The most frequently mutated codon of *ERBB2* was S310 at the furin-like domain (9 of 16 mutated cases, 56.3%), with most of the remaining mutations occurring in the kinase domain and considered oncogenic or likely oncogenic (Table 6). *ERBB3* mutations had a more widespread distribution in the gene with no specific codon position mutated in more than 2 cases. Three of these four genes were also the most commonly amplified genes of the pathways (*KRAS*: 7.4%, *ERBB2*: 14.7%, *ERBB3*: 8.8% of the receptor tyrosine kinase pathway-altered group, Table 6). Notably, *FGFR2* fusions were absent in gallbladder carcinomas, which contrasts with the prevalence of 13.6% (56 of 412 cases) in intrahepatic biliary carcinomas (Table 6).

**Table 6:** List of alterations in receptor tyrosine kinase pathway genes in the MSK gallbladder carcinoma series (*n* = 244)

Gene	Alterations (n) (%)	Mutations (n)		amplifications	fusions
		all	Putative driver		
KRAS	27 (12)	17	17	10	
NRAS	1 (0.4)	1	1		
BRAF	7 (3)	7	7		
PIK3CA	25 (11)	25	24		
EGFR	13 (6)	3	1	8	2
ERBB2	37 (16)	16	14	20	1
ERBB3	26 (12)	14	10	12	
ERBB4	5 (2.1)	5	2		
FGFR1	1 (0.4)			1 deletion	
FGFR2	6 (2.6)	3	1	3	
FGFR3	7 (3)	1	0	4 (1 deletion)	2
FGFR4	4 (1.7)	4	0		
NTRK1	5 (2.1)	3	1	1	1
NTRK2	4 (1.7)	4	1		
NTRK3	5 (2.1)	5	1		
PTEN	11 (5)	7	7	4 deletions	
AKT1	2 (0.9)	2	2		
AKT2	6 (2.6)	2	0	1 (1 deletion)	2
AKT3	1 (0.4)	1	0		
all	136 (57.6)	120	89	59 (7 deletions)	8

Clinical analyses included 233 unique patients and 11 patients contributed 2 samples. The two groups of gallbladder cancers with and without receptor tyrosine kinase pathway mutations did not differ significantly in age, gender or tumor grade (Table 7). High TMB cancers (above 10 mutations/Mb) were more frequent in patients with receptor tyrosine kinase pathway mutations (16.9%) compared with those without such mutations (5%, difference between the two groups 11.9%, 95% confidence interval: 3.5%−18%, Fisher’s exact test *p* = 0.007). Chromosomal instability (CIN) high tumors, as defined by a FGA above 0.1, were also more frequently observed in the group with receptor tyrosine kinase pathway mutations (50%) than without mutations (40%), but this difference did not reach statistical significance (Fisher’s exact test *p* = 0.14, Table 7). The cutoff of 0.1 for FGA was chosen arbitrarily as it is one of the frequently used cutoffs and defines about one third of gallbladder carcinomas as high CIN, which is associated with inferior survival outcomes [19, 20].

**Table 7:** Clinical, pathologic and genomic characteristics of the entire MSK gallbladder carcinoma cohort and of the groups with and without alterations in receptor tyrosine kinase pathway genes

	All (n = 233 patients and *n* = 244 samples)(%)	Patients with receptor tyrosine kinase pathway gene mutations (*n* = 136) (%)	Patients without receptor tyrosine kinase pathway gene mutations (*n* = 100) (%)	p
Age (mean)	65.2 ± 11.3	65.2 ± 11.6	65.6 ± 10.9	0.78
Early onset (≤ 50 years-old)				
yes	28 (11.8)	16 (12.1)	9	0.52
no	210 (88.2)	116 (87.9)	89	
NA	6	4	2	
Gender				
Male	73 (31.3)	40 (29.4)	34 (34)	0.47
Female	160 (68.7)	96 (70.6)	66 (66)	
Grade				
Well/Moderately differentiated	110 (47)	67 (51.1)	40 (41.7)	0.17
Poorly differentiated	124 (53)	64 (48.9)	56 (58.3)	
NA	10	5	4	
TMB				
High (> 10 mutations/Mb)	28 (11.5)	23 (16.9)	5 (5)	0.007
Low (≤ 10 mutations/Mb)	216 (88.5)	113 (83.1)	95 (95)	
FGA				
< 0.1	134 (54.9)	68 (50)	60 (60)	0.14
> 0.1	110 (45.1)	68 (50)	40 (40)	

The cohort included 233 patients with 244 samples as more than one sample was available for a few patients. *TMB*: Tumor Mutation Burden, *FGA*: Fragment Genome Altered, *NA*: Not available

**Fig. 7:**
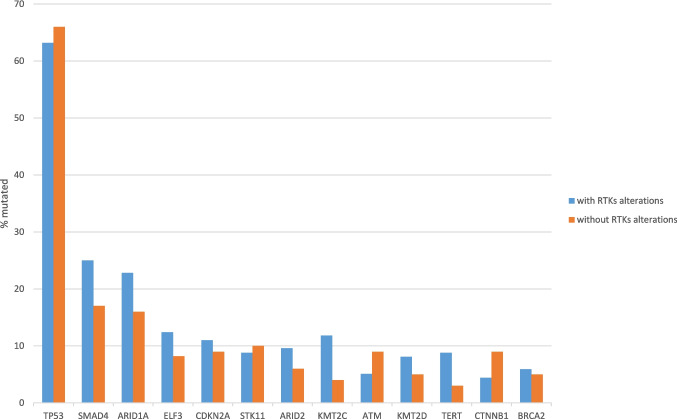
Prevalence of mutations in gallbladder carcinomas with (blue bars) and without (orange bars) receptor tyrosine kinase alterations. Data are from the MSK gallbladder carcinoma series

**Fig. 8:**
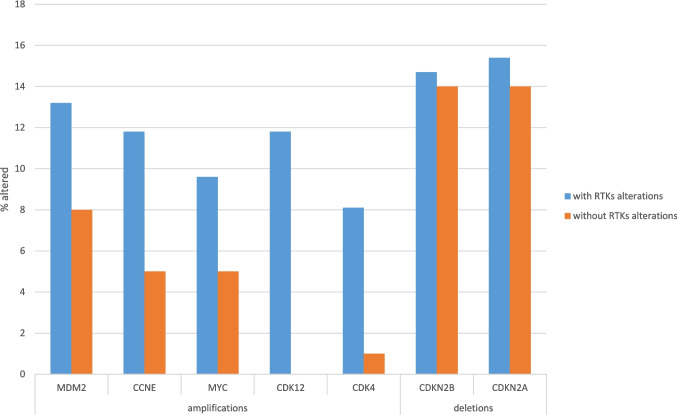
Prevalence of copy number alterations in gallbladder carcinomas with (blue bars) and without (orange bars) receptor tyrosine kinase alterations. Data are from the MSK gallbladder carcinoma series

Mutations in *TP53*, which were more frequent in gallbladder carcinomas (63.1%, 154 of 244 cases) than in intrahepatic cholangiocarcinomas (17%), had a similar prevalence in gallbladder cancers with and without receptor tyrosine kinase pathway mutations (63.2% versus 66%, respectively, Fisher’s exact test *p* = 0.68, Fig. 7). The second most frequent mutations in gallbladder carcinomas, besides receptor tyrosine kinase pathway mutations, were in *SMAD4* (21.3%) and they had also similar prevalence in the groups with or without receptor tyrosine kinase pathway mutations (25% versus 17%, Fisher’s exact test *p* = 0.15, Fig. 7). SMAD4 loss has been associated with lymphovascular invasion which is an adverse prognostic feature [21]. Similar rates of mutations in the two groups were also observed in the epigenetic modifier gene *ARID1A* and other genes with lower rates of mutations overall in gallbladder cancers (Fig. 7). The only exception was the gene encoding for methyltransferase *KMT2C*, which was mutated in 11.8% of cases with receptor tyrosine kinase pathway mutations and in 4% of cases without such mutations (Fisher’s exact test *p* = 0.03, Fig. 7). This difference was, however, not significant after correction for multiple comparisons. KMT2C loss may confer PARP inhibitor sensitivity. Regarding copy number alterations, cases with receptor tyrosine kinase pathway mutations had a higher rate of amplifications in frequently amplified loci than the group without receptor tyrosine kinase pathway alterations. These were statistically significant for two cyclin dependent kinases, the transcriptional kinase *CDK12* (amplified in 11.8% of cases with receptor tyrosine kinase pathway alterations and in no cases in the group without receptor tyrosine kinase pathway alterations) and the cell cycle kinase *CDK4* (amplified in 8.1% of cases with receptor tyrosine kinase pathway alterations and in 1% of cases in the group without receptor tyrosine kinase pathway alterations) at chromosome locations 17q12 and 12q14.1 (Fisher’s exact test *p* = 0.0001 and *p* = 0.01, respectively, Fig. 8).

### Comparison of the Genomic Landscapes of Biliary Cancers According to Primary Locations

**Fig. 9:**
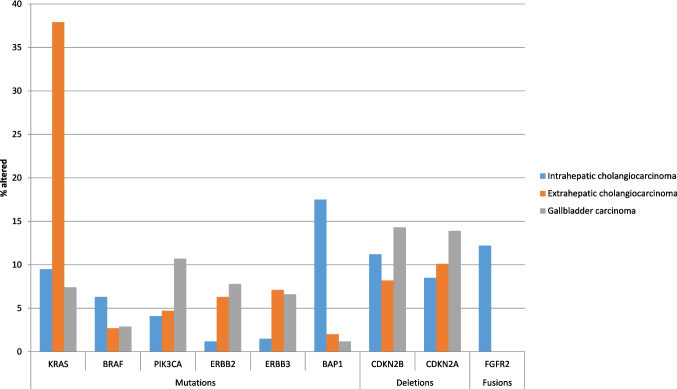
Comparison of the prevalence of frequent mutations in intrahepatic, extrahepatic and gallbladder carcinomas

Although biliary tract cancers have been traditionally treated as a single type of cancer irrespective of primary location along the biliary tree, genomic studies have recently clarified a diverse genomic landscape. The focus of the current analysis, presented in the previous sections, was on the alterations of receptor tyrosine kinase pathways and has disclosed differences in the overall prevalence of these alterations and significant differences of individual genes of the pathways. Several individual genes encoding for receptor tyrosine kinases and the pathways triggered by them have high, albeit variable, frequency of alterations in biliary cancers of intrahepatic, extrahepatic and gallbladder origin. *KRAS* mutations have a high prevalence in extrahepatic cholangiocarcinomas, in which about two out of five cases (37.9%) bear mutations, while intrahepatic (9.5%) and gallbladder carcinomas (7.4%) showed a lower prevalence of *KRAS* mutations (Fig. 9). The targetable *KRAS* G12C mutations were observed in 7.7% of intrahepatic carcinoma *KRAS* mutant cases, 11.1% of *KRAS* mutant gallbladder carcinomas, and in 4.1% of extrahepatic *KRAS*-mutated tumors.

*BRAF* mutations had a low overall prevalence in biliary cancers, but they were more frequent in intrahepatic cholangiocarcinomas (7%) and showed a lower prevalence in gallbladder and extrahepatic carcinomas (about 3%, Fig. 9). Alterations in the gene encoding for receptor HER2, *ERBB2* were rare in intrahepatic carcinomas, in which amplifications were observed in 1.5% of cases. Extrahepatic cholangiocarcinomas showed a higher frequency of *ERBB2* alterations (13.5%), which were shared between mutations (6.3%) and amplifications (7.2%). Gallbladder carcinomas had also a high frequency of *ERBB2* alterations (16%) and, similarly to extrahepatic cholangiocarcinomas, both mutations and amplifications were present. High ERBB2 alteration rates in extrahepatic cholangiocarcinomas and gallbladder carcinomas support HER2-targeted strategies in these cancers (See subsequent section on Current Targeted Options). *FGFR2* fusions were observed exclusively in intrahepatic cholangiocarcinomas, where their frequency was 13.6%, with no cases in the gallbladder cancer cohort. The extrahepatic cohort of the project GENIE contains no assessment of the presence of fusions, but another small series that included 36 extrahepatic cholangiocarcinoma samples confirmed no *FGFR2* fusions in these cases [22]. Therefore, *FGFR2* fusions are intrahepatic carcinoma-specific defining a distinct therapeutic subset. Embryologic provenance differences in stem cell origins or carcinogen exposure may drive *FGFR2* fusion specificity.

Besides receptor tyrosine kinase pathways, mutations in the tumor suppressor *TP53* showed a higher rate in gallbladder carcinomas, around 60%, with a moderately lower prevalence in extrahepatic cholangiocarcinomas at 50%, while intrahepatic cholangiocarcinomas displayed a significantly lower prevalence (17%). *TP53* mutation frequency differed significantly between subtypes (χ2 test *p* < 0.0001). Conversely, *CDKN2A*/*CDKN2B* losses were frequent in intrahepatic receptor tyrosine kinase pathways -altered tumors (*p* < 0.001) but rare elsewhere. Moreover, intrahepatic cholangiocarcinomas displayed *IDH1* mutations in 20.6% of cases and an even higher prevalence (25%) in those without receptor tyrosine kinase pathways mutations and *IDH1*/*IDH2* mutations inversely correlated with receptor tyrosine kinase pathway alterations (OR: 0.45, 95% CI 0.3–0.7; *p* < 0.001), while *IDH1* mutations were rare (2.3%) in extrahepatic cholangiocarcinomas and absent in gallbladder carcinomas. *IDH2* mutations also occurred in intrahepatic cholangiocarcinomas only, although less common (4.4%) and, similarly to *IDH1* mutations, more frequently (7.1%) in cases without receptor tyrosine kinase pathways mutations. Another prevalently mutated gene in intrahepatic cholangiocarcinomas, *BAP1* (BRCA1 associated protein 1, 17.5%) did not show differential prevalence in cases with or without receptor tyrosine kinase pathways mutations. *BAP1* mutations were rare (1.2% to 1.7%) in extrahepatic cholangiocarcinomas and gallbladder carcinomas. *BAP1* loss may confer synthetic lethality with PARP inhibitors, as will be discussed in the next section. *ARID1A* mutations were common in all three biliary cancer locations with a prevalence of 17% to 20%. Another epigenetic modifier and SWI/SNF member, *PBRM1* was mutated in 10% of intrahepatic cholangiocarcinomas and in 3% to 4% of extrahepatic cholangiocarcinomas and gallbladder carcinomas. *SMAD4* mutations trend higher in gallbladder versus extrahepatic carcinomas (21.3% versus 15.7%, *p* = 0.12). Gallbladder receptor tyrosine kinase pathway-altered tumors uniquely associate with high TMB (*p* = 0.007), potentially influencing immunotherapy response.

## Discussion

### Current Targeted Therapeutic Options

Several targeted therapeutic options exist for biliary cancer patients in the group with receptor tyrosine kinase pathway gene alterations. Individual alterations in several of these genes may be targeted with currently available drugs (Table 1). Several such drugs have been approved and have been incorporated in practice guidelines [8, 23]. FGFR inhibitors, such as futibatinib, and pemigatinib, have been approved for the treatment of patients with FGFR fusions (Table 8) [24–26]. These fusions and other FGFR family gene alterations (as well as *IDH1*/*IDH2* mutations) appear to confer improved survival in biliary cancers [27]. FGFR-altered tumors showed longer OS (HR 0.42, 95% CI: 0.21–0.87, *p* = 0.003) in chemotherapy-treated patients [27]. Patients developing progression on FGFR inhibitors bear frequently new FGFR2 mutations or mutations in the pathways downstream [28]. Resistance involves *FGFR2* kinase domain mutations (N540K, V565F), bypass signaling (MET/RAS), or phenotypic shifts [28, 29]. An in vitro study in biliary cancer cells showed that cells with FGFR2-BICC1 fusions were sensitive to FGFR inhibitors, while concomitant *KRA*S G12D or *BRAF* V600E mutations bestowed resistance to these drugs, suggesting that a wider panel of molecular alterations could be informative as a predictive biomarker of response [28]. New kinase domain mutations in FGFR2 have also been observed in intrahepatic cholangiocarcinoma patients treated with erdafitinib and developing progression [29]. Up-regulation of PI3K/AKT signaling was present after the development of resistance and was overcome by adding EGFR or mTOR inhibitors. In the clinic, besides additional mutations in gatekeeper and molecular brake sites on FGFR2, low drug concentrations have also been identified as associated with development of resistance [30]. FGFR inhibitors are now moving to first line therapy in biliary cancers with FGFR2 alterations. For example the FIGHT-302 phase 3 trial (NCT03656536) examines pemigatinib versus gemcitabine/cisplatin chemotherapy in the first line treatment of patients with FGFR2 fusions or rearrangements [31].

**Table 8:** Trials of receptor tyrosine kinase pathways targeted therapies in advanced/metastatic biliary cancers

Reference	Phase	Number of pts and line of treatment	Drugs	Alteration targeted and cut-off	Outcomes	Adverse effects
[24]	2	122 (108 with FGFR2 fusions or rearrangements), second or later line	Infigratinib	FGFR2 fusions or rearrangements	ORR; 23.1% (95% CI: 15.6%- 32.2%)	Hyperphosphatemia, stomatitis, fatigue, alopecia, dry eyes, central serous retinopathy
[25]	2	146 (107 with FGFR2 fusions or rearrangements), second or later line	Pemigatinib	FGFR2 fusions or rearrangements	ORR; 35.5% (95% CI: 26.5%- 45.4%)	Hyperphosphatemia, stomatitis, arthralgia, hyponatremia
[26]	2	103 with intrahepatic cholangiocarcinoma, second or later line	Futibatinib	FGFR2 fusions or rearrangements	ORR; 42% (95% CI: 32%- 52%)	Hyperphosphatemia, stomatitis, transaminitis, fatigue
[33]	2 basket	43 biliary cancer patients with BRAF V600E mutations, second or later line	Dabrafenib and trametinib	BRAF V600E mutations	ORR: 47% (95% CI: 31%–62%)	Pyrexia, γ-glutamyl-transferase elevations
[34]	2 basket	4 intrahepatic cholangiocarcinoma patients	Dabrafenib and trametinib	BRAF V600E mutations	ORR: 75%	Pyrexia
[37]	2	90 biliary cancer patients (mostly gallbladder) with HER2 positive disease, first line	Gemcitabine and cisplatin with trastuzumab	HER2 positive (3 + by IHC or 2 + by IHC and positive FISH)	ORR: 55.5%, mOS: 10 months (95% CI: 9.2–10.7 months)	Anemia, neutropenia, nausea/vomiting, fatigue, infection
[38]	2	32 biliary cancer patients, 22 patients had HER2 positive disease, second or later line	Trastuzumab deruxtecan	HER2 positive (3 + by IHC or 2 + by IHC and positive FISH)	ORR: 36.4% (95% CI: 17.2%−59.3%) in HER2 positive, mOS: 7.1 months (95% CI: 4.7–14.6 months)	Anemia, neutropenia, interstitial lung disease
[39]	2 basket	29 biliary cancer patients, HER2 or HER3 positive disease, second or later line	Trastuzumab and pertuzumab	HER2 positive (3 + by IHC or 2 + by IHC and positive FISH) or mutated or HER3 alterations	ORR: 32% (95% CI: 16%−52%)	Anemia, diarrhea, fatigue
[40]	2	34 biliary cancer patients with HER2 positive disease, second or later line	FOLFOX/trastuzumab	HER2 positive (3 + by IHC or 2 + by IHC and positive FISH)	ORR of 29.4% (95% confidence interval: 16.7%−46.3%)	Anemia, neutropenia, peripheral neuropathy
[41]	2 basket	30 biliary cancer patients with HER2 positive disease, second or later line	Trastuzumab and tucatinib	HER2 positive (3 + by IHC or 2 + by IHC and positive FISH) or mutated or HER3 alterations	ORRl 46.7% (90% CI: 30.8%−63%)	Pyrexia, diarrhea, infection, inappetence

*ORR*: Objective Response Rate, *CI*: Confidence Interval, *mOS*: median Overall Survival.

*BRAF* mutated biliary carcinomas have worse prognosis than biliary carcinomas without mutated *BRAF* [32]. The biliary cancer cohort of the phase 2, open label ROAR (Rare Oncology Agnostic Research) trial included 43 patients with BRAF V600E mutations who were treated with the BRAF inhibitor dabrafenib and the MEK1/2 inhibitor trametinib [33]. The independent review ORR was 47% (95% confidence interval: 31%–62%) and toxicities were expected and manageable. The median PFS was 9 months and median OS was over a year (13.5 months). The combination of dabrafenib and trametinib was also used in the sub-protocol of the NCI-MATCH basket trial for patients with *BRAF* mutations [34]. Thirty-five patients were enrolled in the sub-protocol, including four patients with intrahepatic cholangiocarcinoma. Three of these four patients had long partial responses lasting 9.1 months, 12.8 months and 29.4 months [34]. Overall, these two trials have shown significant activity of BRAF inhibitor/MEK inhibitor combinations in biliary cancers with BRAF mutations (ROAR: ORR 47% (20 of 43 biliary patients) and NCI-MATCH: 3 partial responses in 4 intrahepatic patients). In another *BRAF* mutated cohort from a basket trial in Japan with a total of 50 patients, six biliary cancer patients were included and four patients were evaluable for response [35]. One of two evaluable patients with intrahepatic cholangiocarcinomas showed a partial response and the two patients with extrahepatic and gallbladder cancers showed stable disease. The combination of dabrafenib and trametinib has been approved, based on these results for *BRAF* mutated cancers, including biliary cancers [36].

HER2 blockade in *ERBB2* amplified and mutated biliary cancers has been evaluated as a therapeutic strategy [37–41]. In the first line setting, trastuzumab in combination with gemcitabine and cisplatin was associated with an overall response rate (ORR) of 55.5% and disease control rate (DCR) of 80% in a phase 2 trial with 90 HER2 positive metastatic biliary cancer patients [37]. Included patients had predominantly gallbladder primaries (96%). Median progression free survival (PFS) was 7 months (95% confidence interval: 6.2 months to 7.8 months) and the PFS at 6 months was 75.6% (95% confidence interval: 66.6% to 84.6%). In a phase 2 single arm study in patients with HER2 positive, unresectable or refractory biliary cancers and progression or intolerance on gemcitabine based chemotherapy, treatment with the antibody drug conjugate trastuzumab deruxtecan produced an ORR of 36.4% (95% confidence interval: 17.2%−59.3%) [38]. A few patients who had HER2 low disease were also included and had an ORR of 12.5% [38]. The approach of combining trastuzumab with pertuzumab was used in one of the arms of the TAPUR basket trial in patients with biliary cancers and alterations (amplifications, over-expression or mutations) of *ERBB2* or *ERBB3* [39]. The ORR with the combination was 32% (95% confidence interval: 16%−52%) and DCR was 40% (90% confidence interval: 27%−100%), both meeting the pre-specified criteria for activity [39]. In the second or third line treatment of patients with metastatic biliary cancers and ERBB2 amplification or over-expression progressing on gemcitabine and cisplatin, a phase 2 study disclosed an ORR of 29.4% (95% confidence interval: 16.7%−46.3%) and DCR of 79.4% (95% confidence interval: 62.9%−89.9%) with FOLFOX/trastuzumab treatment [40]. Median PFS and OS was 5.1 (95% confidence interval: 3.6–6.7 months) and 10.7 months (95% confidence interval: 7.9 months to not reached), respectively. In a trial of pretreated HER2 positive biliary cancer patients who had not received previous HER2 targeting therapies, 30 patients were treated with the combination of trastuzumab with tucatinib [41]. The ORR obtained with this treatment was 46.7% (90% confidence interval: 30.8%−63%) and DCR was 76.7% (90% confidence interval: 60.6%−88.5%). Overall, the evidence from these non-randomized studies show significant activity from targeting HER2 in biliary cancers, but the optimal management and sequence of anti-HER2 therapies need to be confirmed preferentially in a randomized trial. In addition, targeted treatments may also have significant toxicities that may also become lethal in some patients, such as the risk of pneumonitis with trastuzumab deruxtecan, necessitating close monitoring and expert management [38]. It is worth noting here regarding the adverse effect profile of other targeted drugs discussed above, that although are well tolerated overall, FGFR inhibitors do require monitoring for hyperphosphatemia and ocular toxicity [24–26]. In addition, BRAF/MEK inhibitors commonly cause fever and rash [33].

Beyond receptor tyrosine kinase pathways, IDH mutations may be targeted with the oral inhibitor ivosidenib, which has been approved for the second or third line treatment of patients with metastatic biliary cancers bearing IDH1 mutations [42]. In a phase 3 randomized placebo control trial, ivosidenib treatment showed a median OS of 10.3 months in these pretreated patients, which was statistically significantly better compared with placebo, when adjusted for cross-over (HR 0.69, 95% CI: 0.56–0.84, *p* < 0.001). In addition, the drug was well tolerated with no worsening in quality of life levels [42].

**Fig. 10:**
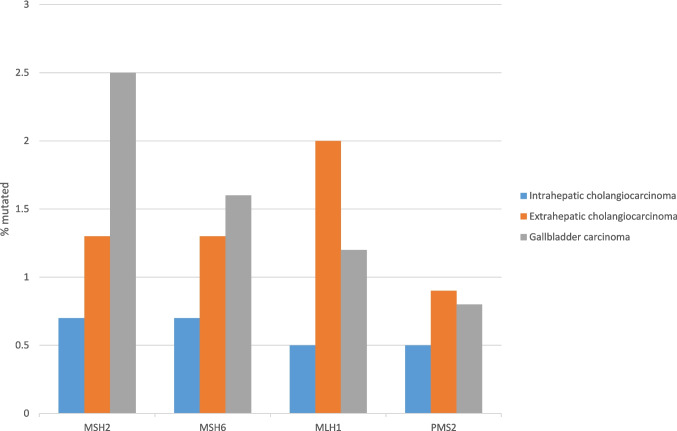
Comparison of the prevalence of mutations in mismatch repair genes (MMR) in intrahepatic, extrahepatic and gallbladder carcinomas

Immunotherapy with PD-1 inhibitors durvalumab or pembrolizumab in combination with chemotherapy has been added to the standard first line therapy of metastatic biliary carcinomas, without consideration of biomarkers or location [43–45]. The addition of durvalumab has improved the 2 year OS of metastatic previously untreated biliary cancer patients to 24.9% (95% confidence interval: 17.9%−32.5%) from 10.4% (95% confidence interval: 4.7%−18.8%) with cisplatin and gemcitabine alone [45]. Specific biomarkers predictive of response to immunotherapy, such as MMR deficiency are rare in biliary cancers. Loss of expression of MSH2, MSH6, MLH1 or PMS2 was observed in 3.8% of patients in a series of 286 patients with biliary cancers treated in a center in Italy [27]. The prevalence of mutations in the series from MSK and project GENIE in each of the MMR related genes varied between 0.5% and 2.5% (Fig. 10). In addition, high TMB (above 10 mutations/Mb) was observed in 4.9% of intrahepatic cholangiocarcinomas, while extrahepatic cholangiocarcinomas, and gallbladder carcinomas had a higher rate of high TMB at 12.8% and 11.5%, respectively. TMB above 10 mutations/Mb is exploratory as a cut-off and may not be the optimal cut-off for defining benefit from immunotherapy in different cancer primaries. As a comparison the other biomarker of immunotherapy response, PD-L1 CPS with a cut-off above 1% showed ORR of 13% in the KEYNOTE-158 trial biliary cohort. However, most of the cases with high TMB have a TMB just above the cut-off and only few have a TMB above 20 mutations/Mb, a level that increases more significantly the probability of responding to immunotherapy. A higher density of tumor infiltrating lymphocytes (TILs) in biliary tumor microenvironment portends a good prognosis and may also be predictive of response to immune checkpoint inhibitors [46, 47]. In a study that used artificial intelligence to assist in determining TILs number and location inside or in the periphery of tumors, 11.8% of metastatic biliary cancer patients had an inflamed immune phenotype with high TIL numbers inside the tumor [47]. These patients showed an overall response rate (ORR) of 27.5% to second or later line immune checkpoint inhibitors, while patients with immune excluded tumors or immune-desert pattern tumors has an ORR of 7.7%. Therefore, inflamed tumors may have a higher response rate to immunotherapy than tumors with tumors with a high TMB, defined as more than 10 mutations/Mb or tumors with PD-L1 CPS above 1%, although direct comparisons are lacking. Median OS was also significantly longer in patients with inflamed immune phenotype (12.6 months versus 5.1 months in non-inflamed patients, HR: 0.46, 95% confidence interval: 0.29–0.73, *p* < 0.001). The available data imply that a combination of available biomarkers could provide an improved predictive power for determining benefit from immunotherapy in biliary cancers and may capture a broader group of patients who will have a significant benefit from these therapies. However, the value of multimodal biomarkers requires validation.

### Future Targeted Therapeutic Perspectives

After progression on first line combination chemotherapy with immune checkpoint inhibitor immunotherapy, second line therapy for most biliary cancer patients with no targeted options include second line combination chemotherapy [48, 49]. These treatments induce a response rate in about 10% of patients and are associated with a median overall survival of 6 to 7 months [50, 51]. Hence, additional effective targeted therapies could be of significant clinical value. The randomized open label phase 2 Vecti-BIL trial (89 chemotherapy naïve, *KRAS* wild type advanced biliary cancer patients) from Italy has not shown benefit of adding panitumumab to gemcitabine/oxaliplatin (GEMOX) [52]. The median PFS was 5.3 months (95% CI: 3.3–7.2 months) with GEMOX/panitumumab and 4.4 months (95% CI: 2.6–6.2 months) with GEMOX alone. OS was 9.9 and 10.2 months, respectively. A non-significant trend for a benefit with panitumumab was observed in intrahepatic cholangiocarcinoma (15.1 versus 11.8 months, *p* = 0.13). The intrahepatic type of biliary cancers possess the lowest percentage of collective mutations in receptor tyrosine kinase pathway genes (about 50% compared with 60%−65% in the other primaries), the absence of which may improve their sensitivity to EGFR inhibitors. Therefore, screening for a broader panel of alterations in the pathway, such as the one used for decision making in colorectal cancers could be of benefit in biliary cancer patients. However, prospective randomized evaluation would be required to confirm if biliary cancer patients without a broader spectrum of receptor tyrosine kinase pathway alterations would benefit from the addition of anti-EGFR antibodies to chemotherapy. Moreover, better patient selection through detailed evaluation of receptor tyrosine kinase pathway alterations may help pinpoint to patients that would remain sensitive to anti-EGFR drugs even in the post-chemoimmunotherapy setting, which constitutes currently the established first line choice. Additional receptor tyrosine kinase targets present in sub-sets of biliary cancers such as *ERBB3* alterations which are observed in 12% of gallbladder carcinomas warrant exploration, for example with the HER3-targeted antibody–drug conjugate patritumab deruxtecan. Biliary cancers with receptor tyrosine kinase pathway alterations and high TMB or TIL infiltration could be targets of combinations of targeted agents with immunotherapy.

Targeting other receptor tyrosine kinase pathway components in biliary cancers with the respective alterations is also of therapeutic interest. PI3K kinase inhibitors in cancers with *PIK3CA* mutations, possibly in combination with other inhibitors of the pathways, may provide superior efficacy over monotherapy by providing a more complete inhibition and preventing development of resistance through activation of feedback loops operating in the network [53]. A preclinical study in cholangiocarcinoma cells and xenografts found that the combination of PI3K inhibitor buparlisib and the multikinase inhibitor ponatinib, which has activity against FGFR, VEGFR, PDGFR and the chronic myeloid leukemia bcr-abl fusion kinase, was more effective than monotherapy [53]. This study did not examine whether alterations in the targeted proteins were present in the cell lines used. Another study suggested that IGF-1R inhibition may be critical for suppressing cancer stem cell populations from cholangiocarcinomas [54]. A phase 2 clinical trial of the PI3K inhibitor copanlisib in combination with cisplatin and gemcitabine as first line therapy of metastatic biliary cancer patients did not improve PFS compared with historical controls [55]. Median PFS was 6.2 months and median OS was 13.7 months. In an exploratory analysis, low expression of phosphatase PTEN was associated with longer PFS (median 8.5 months) and OS (median 17.9 months) than the PFS and OS in patients with high PTEN expression (median PFS: 4.6 months and median OS: 7 months, *p* = 0.19 for both comparisons). The exploratory PTEN expression findings require prospective confirmation. These data imply that even when combined with chemotherapy which has non-specific target effects, biomarkers would be required for optimal use of targeted therapies.

Specific inhibitors of G12C KRAS mutations have been approved for the treatment of lung cancer, where this mutation is most prevalent and are also studied in other cancers [56]. Twelve biliary carcinoma patients were included in the phase 2 trial of the inhibitor adagrasib [57]. Most patients had received previous gemcitabine and fluoropyrimidine-based lines of chemotherapy. The ORR of the biliary cohort was 41.7% (95% CI: 15.2% to 72.3%), median PFS was 8.6 months (95% CI: 2.7 months to 11.3 months) and median OS was 15.1 months (95% CI: 8.6 months to not evaluable). Therefore, despite modest G12C prevalence, adagrasib shows promising activity. As detailed above, G12C mutations are less frequent in biliary carcinomas (7.7% of intrahepatic carcinomas with *KRAS* mutant cases, 11.1% of *KRAS* mutant gallbladder carcinomas, and 4.1% of extrahepatic *KRAS*-mutated tumors). Biliary tract cancers possess G12D mutations, as their most prevalent *KRAS* mutation. Inhibitors of this specific mutation have entered clinical trials and they would be useful in a greater spectrum of biliary cancer patients [58]. The combination of G12C KRAS inhibitors with anti-EGFR monoclonal antibodies has been developed as a strategy to prevent resistance in colorectal cancers with these mutations [59, 60]. A similar strategy in biliary cancers could be envisioned and combinations with KRAS inhibitors of other mutations could also be developed for the more common *KRAS* mutations in the disease. A study in *KRAS* mutated intrahepatic cholangiocarcinoma xenografts showed a vulnerability of these models to PARP-1 knockdown [61]. A dependency on PARP1 for the DNA damage response mediated by CHK1 kinase was observed in cholangiocarcinoma cells with oncogenic *KRAS* mutations, suggesting a potential therapeutic role for pharmacologic inhibition of PARP.

BAP1 is a de-ubiquitinase with functions in the regulation of DNA repair and the regulation of apoptosis and ferroptosis [62]. BAP1 is frequently mutated in mesotheliomas and uveal melanomas and is the culprit in a hereditary cancer predisposition syndrome, which includes, besides these two cancers, cutaneous melanomas and renal carcinomas. Although uncommonly mutated in other biliary tumors, with a mutation prevalence of less than 2% in extrahepatic and gallbladder carcinomas, *BAP1* is mutated in 17.5% of intrahepatic cholangiocarcinomas, which is the highest frequency outside the four tumors of the hereditary syndrome. *BAP1* mutations showed equal distribution in tumors with and without receptor tyrosine kinase alterations, making both groups a potential therapeutic target, including the group without such alterations, which has fewer therapeutic options. However, tumor suppressors have been notoriously difficult to target, as this would require reconstituting the function of the mutant protein. Therefore, rather than direct targeting, a synthetically lethal approach, such as the one employed in targeting the partner of BAP1, BRCA1 with PARP inhibitors, is more practical [63]. PARP inhibitors olaparib, veliparib and talazoparib and the ATR inhibitor AZD6738 were effective in inhibiting the survival of a panel of cholangiocarcinoma cell lines, bearing 9 to 15 mutations in 27 genes involved in DNA damage response [64]. Cell lines with more mutations in these genes were more sensitive to the drugs and the combination of AZD6738 with one of the PARP inhibitors was synergistic with more pronounced effects in the less sensitive lines than monotherapies. The ATR and PARP inhibitor synergism in cholangiocarcinoma cell lines with DNA damage response gene mutations provide a rational for BAP1 specific studies in this setting. The KRAS-driven PARP dependency and BAP1 loss PARP inhibitor synthetic lethality remain preclinical observations, while clinical trials have explored IDH1 mutations and PARP inhibitor sensitivity due to the role of IDH mutations in homologous recombination defects. PARP inhibitors have been studied in intrahepatic cholangiocarcinoma patients with IDH1 mutations, given the involvement of the oncometabolite 2-hydroxyglutarate in DNA hypermethylation, leading to homologous recombination defects and a BRCAness phenotype [65]. Forty patients with IDH1 mutations were treated with PARP inhibitors alone or combined with ATR inhibitors or immune checkpoint inhibitors. ORR was 5% and PFS ranged from 1.4 months to 18.5 months [66]. The range of OS was from 2.8 months to 42.4 months, suggesting that, while overall efficacy of PARP inhibitors in this population is low, some patients may benefit.

PBRM1 (polybromo 1, also called SMARCH1 or BAF180) is an epigenetic modifier protein of the SWI/SNF complex, representing, together with ARID1A epigenetic modifiers of this complex commonly mutated in biliary cancers. *PBRM1* mutations, which are observed in about 10% of intrahepatic cholangiocarcinoma and more often in those without receptor tyrosine kinase alterations, may also become targets for synthetic lethal approaches, as they sensitize to PARP inhibitors, although the relevance of these preclinical data in the specific context of biliary cancers is unproven [67].

The gastric isotype of tight junction protein claudin 18.2 is also expressed in cholangioacarcinomas and could be targeted therapeutically with the monoclonal antibody zolbituximab, currently approved in gastric and gastroesophageal junction adenocarcinomas, or with antibody drug conjugates in development [68, 69]. A study, using moderate to strong membrane staining in at least 75% of tumor cells as definition of positivity, found 13.1% of cases in a tissue microarray of 160 cholangiocarcinomas to be claudin 18.2 positive [7]. Perihilar cholangiocarcinomas were more often positive than intrahepatic cholangioacarcinomas. These sub-sets of biliary tumors could be candidates for targeted therapies with monoclonal anti-claudin 18.2 antibodies and appropriate target cases may be expanded with the development of antibody drug conjugates, already in clinical trials in gastric cancers, which may require less strong expression of the target, due to a bystander effect. Anti-claudin 18.2 agents, such as TST001, are in early-phase biliary cancer trials (NCT04495296).

## Conclusions

In conclusion, studies elucidating the genomic making of biliary carcinomas have paved the way for the introduction of targeted therapies in a personalized manner. Priority trial candidates for rational drug development include biomarker-driven trials for BAP1/IDH1 losses, G12C/G12D inhibitors, and claudin 18.2 antibody–drug conjugates. It is hoped that genomic studies will help the development of further rational treatments, based on new and more effective drugs. Besides direct targeting, synthetic lethal approaches based on genomic studies may tackle the treatment of cancers without directly targetable alterations.

## Data Availability

No datasets were generated or analysed during the current study. The authors declare no competing interests.
